# The Inter-Dependence of Diseases of the Teeth and of the Female Pelvic Organs

**Published:** 1870-04

**Authors:** N. W. Hawes

**Affiliations:** Boston. Demonstrator of Operative Dentistry in Harvard University.


### ARTICLE IX.

### *The Inter-dependence of Diseases of the Teeth and of the Female Pelvic Organs.*

By N. W. Hawes, Boston.

Demonstrator of Operative Dentistry in Harvard University.

The reflex influence produced by diseased teeth opens a subject so patent to the Medical and Dental professions, that I feel my inability to inspire new thought upon the universally accepted fact, that disease in an organ may and does excite sympathy in contiguous or remote parts of the physical apparatus. Though the teeth are classed among the "superflous organs," yet in their disease it has been shown that they exert a vital influence upon the whole living system. Among the affections enumerated by Dr. Fitch, in one of his dental works, as occasioned by diseased teeth, are phthisis pulmonalis, dyspepsia, inflammation of the eyes, epilepsy, hysteria, hypochondriasis, rheumatic affections, tic doloreux, etc.; and he asks, in speaking of alarming diseases as being produced by slight causes, "It is unfair, or unreasonable, to suppose that a diseased state of the teeth, or their being in a state of putrefaction and constant irritation and inflammation, should at times produce the most fatal diseases in the general system?"

Now, it is not necessary that they should be in a state of putrefaction to engender diseased influence. I at one time called upon a medical friend suffering from neuralgia, as he said, and remarking that he "was sorely afflicted at times," and had exhausted the whole list of anodynes, and found but temporary relief. I questioned him in regard to his teeth, eliciting the reply, that they were "sound as a nut, every one of them." On my persisting, he suffered me to make an examination, which resulted in the discovery of a left superior bicuspid root entirely covered by a healthy appearing gum. This root was not purulent, or even unhealthy to the eye, but its removal put an end to his neuralgic sufferings, and fully converted him to belief in reflex influences of the teeth. Neither is it necessary that the teeth should be painful, to create disease. Is it uncommon for painless tumours to occasion death; or for foreign and effete matter to produce the same result, even when entirely unsuspected as the cause, until this is developed by autopsy? I could relate several cases where marked and immediate improvement in health has followed the removal of diseased teeth, whose influence has not been suspected. I will cite but one instance. About seven years ago, a lady called upon me for advice respecting her teeth. She had suffered long from dyspepsia, had a hacking cough and hectic fever, was exceedingly nervous, and of course somewhat emaciated. There was not a sound tooth to be found; her gums were inflamed and putrid, with pus exuding from around nearly all her teeth. I at once advised their removal, and the adjustment of an artificial set. She questioned the propriety of going to the expense, inasmuch as her health was so precarious that she did not expect to live long. I dwelt upon the probability of an improvement in the general health after release from her teeth, and finally persuaded her to submit to the operation. The next day she came in and allowed me to extract her teeth,—twenty-eight in all—without anæsthesia, and thus remove the cause of all her infirmities, as was subsequently demonstrated by her speedy return to health. I saw her a few days ago, and she said she had "not been sick a day since I took her teeth away."

Who can doubt the pernicious and even fatal effect of the masses of disease that exist in some mouths, when we consider their contaminating influence over twenty thousand inspirations every twenty-four hours, of heaven's purifier to life itself, the blood, or the numerous nervous disorders that arise from the teeth, too often the primary cause? Is it not startling that the medical profession pay so little attention to the teeth, when they consider that the dental nerves are derived from those usually denominated the superior and inferior maxillary, which are the second and third branches of the fifth pair? Do we not at once perceive the intimate connection between the teeth and the whole body? But I will not extend these remarks. It seems but necessary to call attention to the fact, and it will of itself excite prolific thought.

In reversing the problem, with a few cursory inferences from gynæcology, with regard to the reflex influence produced upon the teeth by an unhealthy uterus, I call to mind the expression of some writer, that every child costs its mother a tooth. Now, whether this trite saying be true or not, I know a mother whose teeth were pronounced past saving by a dentist over twenty years ago; she ceased child-bearing, passed the turn of life, and subsequently I filled her teeth, with the firm conviction that my labor was not lost. My impression is that the uterus plays a more important part in the defection of the female teeth than is generally conceded. Dr. Hall says, "there is scarcely a solid texture or fluid that is not altered from its healthy condition by amenorrhœa." Now, anything that would deplete the blood, or give rise to an unhealthy and vitiated secretion of the fluids of the mouth, must exert a deleterious effect upon the teeth, either by producing inflammation of the gums, or by making direct aggression upon the teeth themselves; and, as the female teeth suffer most, we must hold the uterus responsible for part, at least, of these influences upon them.

After operating, some time since, for a lady, I flattered her with the remark that her teeth were much better than the average. A few months afterwards she called upon me looking rather anæmic. An examination revealed a sad condition of her teeth,—her gums were swollen, turgid, and bleeding at the slightest touch, and her teeth badly decayed particularly at the margin of the gums. I confessed my inability to understand the condition, but inquiry from her husband revealed the fact of a miscarriage, and to this I attributed the erosion of her teeth. Was not my inference correct? Erosion of the teeth is obviously the result of the corrosive menstrua that come into contact with them,—the acid principle being the active agent generally, if not always. I knew a lady, who died from cancer of the uterus, whose teeth during the last few weeks of her life were literally washed away. Now, what caused this abundant secretion of acid, if not the diseased uterus? Would there have been the same secretion had the disease been elsewhere situated? Is not the uterus, when diseased, prone to produce a condition of things favorable for the destruction of the teeth? And is not the uterus in a condition to exert a depraved influence upon the fluids during nearly two months in the year, conforming to the menstrual periods? Does not the offspring of a mother, suffering from any of the innumerable diseases of the pelvic organs, inherit an imperfect general organization, to hand down even to the third and fourth generation? I suppose that a child properly brought into existence, and endowed with an unimpaired vital fluid, might live on like Methuselah, and perhaps forget to die, unless by accident, or another flood. Some one has said that the original impartation of life is from the father, but the development depends upon the mother; and if she be healthy and robust, the child will be so, too, almost regardless of the father's physique. Certainly we know that the child inherits a good or bad set of teeth from the maternal, rather than the paternal parent, and that the teeth are much affected, even where a wet nurse is employed, in conformity with the condition of her teeth. To end this digression, I am one of those who do not consider that the organs of reproduction were ever designed for a source of amusement merely, but for the specific object of replenishing the earth; and I sincerely believe that their abuse is the primary cause of a great part of the disease, contracted or inherited, to which flesh is heir. Would that some competent hand would properly treat this subject for the good of a common humanity! It might disgust a Paul, or shock a Joseph; but let the one exempt from the sins referred to cast the first stone.—*Gynæcological Journal.*

